# Gram-negative bacterial colonizations before bilateral lung transplant. The impact of ‘targeted’ versus ‘standard’ surgical prophylaxis

**DOI:** 10.1186/s12879-024-09199-y

**Published:** 2024-03-13

**Authors:** Sabrina Congedi, Arianna Peralta, Luisa Muraro, Martina Biscaro, Tommaso Pettenuzzo, Nicolò Sella, Silvia Crociani, Arméla Anne-Sabine Tagne, Ida Caregnato, Francesco Monteleone, Elisa Rossi, Gabriella Roca, Silvia Manfrin, Serena Marinello, Maria Mazzitelli, Andrea Dell’Amore, Annamaria Cattelan, Federico Rea, Paolo Navalesi, Annalisa Boscolo

**Affiliations:** 1 Department of Medicine (DIMED), University of Padua, Padua, Italy; 2 Azienda Ospedale - Univerisità Padova, Padova, Italy

**Keywords:** Surgical prophylaxis, Prophylaxis, Antimicrobial stewardship, Lung transplant, Bilateral lung transplant, Antibiotics

## Abstract

**Background:**

Infections are one of the most common causes of death after lung transplant (LT). However, the benefit of ‘targeted’ prophylaxis in LT recipients pre-colonized by Gram-negative (GN) bacteria is still unclear.

**Methods:**

All consecutive bilateral LT recipients admitted to the Intensive Care Unit of the University Hospital of Padua (February 2016–2023) were retrospectively screened. Only patients with pre-existing GN bacterial isolations were enrolled and analyzed according to the antimicrobial surgical prophylaxis (‘standard’ vs. ‘targeted’ on the preoperative bacterial isolation).

**Results:**

One hundred eighty-one LT recipients were screened, 46 enrolled. Twenty-two (48%) recipients were exposed to ‘targeted’ prophylaxis, while 24 (52%) to ‘standard’ prophylaxis. Overall prevalence of postoperative multi-drug resistant (MDR) GN bacteria isolation was 65%, with no differences between the two surgical prophylaxis (*p* = 0.364). Eleven (79%) patients treated with ‘standard’ prophylaxis and twelve (75%) with ‘targeted’ therapy reconfirmed the preoperative GN pathogen (*p* = 0.999). The prevalence of postoperative infections due to MDR GN bacteria was 50%. Of these recipients, 4 belonged to the ‘standard’ and 11 to the ‘targeted’ prophylaxis (*p* = 0.027).

**Conclusions:**

The administration of a ‘targeted’ prophylaxis in LT pre-colonized recipients seemed not to prevent the occurrence of postoperative MDR GN infections.

**Supplementary Information:**

The online version contains supplementary material available at 10.1186/s12879-024-09199-y.

## Introduction

Infections are one of the most frequent complications of lung transplantation (LT) and the most common cause of death during the first year, with a mortality rate up to 37% [1, 2]. Overall prevalence of Gram-negative (GN) infections is annually increasing (4.33/1000 recipient-days) [3–6]. The prevalence of multidrug-resistant (MDR) GN bacteria is around 30% after LT, with an in-hospital mortality six times greater than recipients experiencing GN bacterial infections with no antimicrobial resistances [6, 7]. Long-term exposure to immunosuppression to prevent graft rejection has been recognized as the most relevant risk factor for increasing vulnerability to infections [8–10]. Therefore, even if antimicrobials may promote antimicrobial resistance, these medications remain life-saving medications [11].

The impact of both donor and recipient pre-existing colonizations on the occurrence of post-LT pneumonia and other infections is conflicting and debated [6, 12–16]. A relatively low risk of donor-recipient bacteria transmission (up to 2.9% of cases) has been reported and the presence of donor’s organisms has not been necessarily associated with the occurrence of post-LT pneumonia [17]. On the other hand, it’s known that pre-existing recipient’s GN colonizations are an independent predictor of isolation of MDR GN bacteria after LT, despite not always being responsible for severe clinical conditions [7, 14, 18–23].

The optimal antimicrobial approach for pre-operative GN bacterial colonizations in LT recipients is still unclear. Noteworthy, the potential benefit of a personalized surgical prophylaxis, i.e. ‘targeted’ on preoperative colonizations, is still under discussion. The last guidelines reported conflicting data on the titration of antimicrobial prophylaxis based on previous colonization, while a watchful post-LT microbiological surveillance is always recommended for a prompt identification of infections requiring targeted antimicrobial therapies [15, 21–25].

Therefore, aim of this retrospective observational study, enrolling bilateral LT recipients pre-colonized by GN bacteria, was assessing: *(i)* the overall prevalence of MDR GN bacteria, over the whole bacterial isolates, within the first 30 days following bilateral LT; *(ii)* the prevalence of infections and colonizations due to MDR GN bacteria; and *ii)* the impact on short- and mid-term outcomes of the exposure to ‘standard’ or ‘targeted’ surgical prophylaxis (according to in vitro susceptibility).

## Materials and methods

**Fig. 1:**
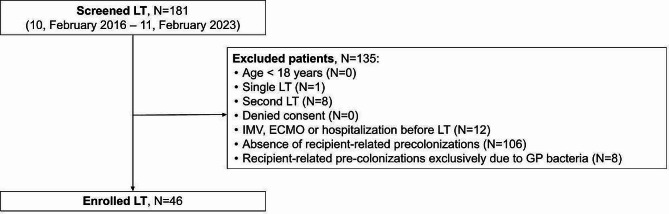
Flow-chart. *Abbreviations*: LT, lung transplant; IMV, invasive mechanical ventilation; ECMO, extracorporeal membrane oxygenation; GP, Gram-positive

The study was approved by the Institutional Ethic Committee of Padua University Hospital (reference number 0025364) and was conducted in accordance with the principles of Good Clinical Practice and according to the Declaration of Helsinki. Written informed consent was obtained from all subjects and/or their legal representatives. The article was written in accordance with the “strengthening the reporting of observational studies in epidemiology-STROBE” checklist (Table S3) [26].. All consecutive patients admitted to our ICU at the Padua University Hospital after the first bilateral LT, between February 10th, 2016 and February 11th, 2023, were retrospectively evaluated and enrolled according to the following inclusion criteria: (1) age > 18 years; (2) written informed consent; (3) absence of invasive mechanical ventilation (IMV), extracorporeal membrane oxygenation (ECMO) and hospitalization before surgery; (4) documented pre-existing recipient-related GN bacterial isolations (Fig. 1). Patients underlined single or a second LT or exclusively pre-colonized by Gram-positive (GP) bacteria were excluded.

All screened LT recipients had at least one complete microbiological screening performed 6 months before surgery and all donor- and recipient-related pre-colonizations were confirmed by biological fluid samples collected perioperatively.

Standardized protocols for perioperative antibiotic management and immunosuppressant therapy were developed in our center following international recommendations and were previously published [6, 18, 21–23]. Specifically, the ‘standard’ surgical prophylaxis included intravenous piperacillin-tazobactam or ceftazidime, plus teicoplanin for GP bacteria. This strategy was followed from October 2020 until February 2023 and applied to all precolonized LT recipients without signs of sepsis or septic shock before LT. By contrast, the ‘targeted’ protocol was applied in a previous period of time (from January 2016 until September 2020) and allowed to use perioperative ‘targeted’ antibiotics, according to the preoperative bacterial isolates, and in vitro susceptibility testing following the European Committee on antimicrobial susceptibility testing (EUCAST) recommendations, in clinically stable LT recipients without signs of sepsis or septic shock before LT [27–32]. After ICU admission, a standardized protocol for microbiological surveillance has been constantly applied in our center service, until hospital discharge, as described in Table S4 and previously published [14]. Specifically, according to our surveillance protocol, we routinely collected, usually at the ICU admission and then every 2–3 days, bronchoalveolar lavage (BAL) and/or bronchoalveolar aspiration (BASP), blood and urine samples (expecially in case of infection), and rectal swabs (Table S4).

Gram-negative bacteria were classified as multi-sensitive (MS) or MDR according to the internationally recognised definitions [33–37]. Conventionally, our ‘MDR’-group included carbapenem-resistant Enterobacterales (CRE), ‘difficult-to-treat’ *Pseudomonas aeruginosas* and carbapenem-resistant *Acinetobacter baumanii* (CRAB) [33–37].

The diagnosis of infection was clinically made according to the definition proposed by “The Third International Consensus Definitions for Sepsis and Septic Shock (Sepsis-3)” and, microbiologically, according to the definitions provided by “CDC/NHSN Surveillance Definitions for Specific Types of Infections”, as previously published [25, 38]. In case of bacteria isolation without (a) signs and symptom of infection, and (b) no meeting the microbiological criteria for infection, as described above, the patient was defined as colonized.

All variables collected from electronic health records were listed in Tables 1, 2 and 3 and S1 and S2.

**Table 1:** Baseline patient characteristics according to surgical prophylaxis exposure

	Overall *N* = 46 (100)	Standard prophylaxis *N* = 24 (52)	Targeted prophylaxis *N* = 22 (48)	*p*-value
Baseline characteristics				
Age, years	43 [33–52]	43 [35–50]	43 [31–55]	0.836
Male gender, *n* (%)	24 (52)	16 (67)	8 (36)	0.075
BMI, kg/m^2^	21 [18–24]	21 [19–24]	20 [18–24]	0.812
Corticosteroids, *n* (%)	23 (50)	11 (46)	12 (55)	0.768
O_2_ therapy, *n* (%)	38 (83)	21 (88)	17 (77)	0.451
Diabetes, *n* (%)	11 (24)	6 (25)	5 (23)	0.999
LAS	35 [34–39]	35 [34–39]	35 [33–39]	0.562
Oto score	3 [1–5]	3 [1–5]	2 [2–6]	0.827
Underlying diseases				
Septic^a^, *n* (%)	30 (65)	15 (63)	15 (68)	0.763
Interstitial^b^, *n* (%)	7 (15)	4 (17)	3 (14)	0.999
Obstructive^c^, *n* (%)	8 (17)	4 (17)	4 (18)	0.999
Others^d^, *n* (%)	1 (2)	1 (4)	0 (0)	-
Previous colonization				
Recipient-related MS GN bacteria	24 (52)	13 (58)	9 (45)	0.395
Recipient-related MDR GN bacteria	22 (48)	11 (42)	13 (55)	0.395
Donor-related GN bacteria^e^	13 (28)	8 (33)	5 (23)	0.521
Recipient-related viral colonization	2 (4)	0 (0)	2 (9)	0.476

Data are expressed as number and (percentage) or median and [interquartile range]. ^a^Septic: cystic fibrosis, bronchiectasis; ^b^Interstitial: idiopathic pulmonary fibrosis, allergic extrinsic alveolitis, non-specific interstitial pneumonia, fibrosing emphysema, lymphocytic interstitial pneumonia, respiratory bronchiolitis interstitial lung; ^c^Obstructive: chronic obstructive pulmonary disease; ^d^Others: idiopathic pulmonary hypertension, veno-occlusive disease, connective tissue disease, α1-anti-trypsin deficiency, lymphangioleiomyomatosis, histiocytosis, sarcoidosis, graft versus host disease. *Abbreviations*: BMI, body mass index; *n*, number; O2, oxygen; LAS, lung allocation score; MS, multisensitive; GN, Gram-negative; MDR, multidrug-resistant; LT, lung transplant;

**Table 2:** Intraoperative characteristics according to surgical prophylaxis exposure

	Overall *N* = 46 (100)	Standard prophylaxis *N* = 24 (52)	Targeted prophylaxis *N* = 22 (48)	*p*-value
**Intraoperative characteristics**				
Time of LT, minutes	440 [348–490]	448 [368–490]	430 [339–486]	0.286
Time of graft ischemia, minutes	608 [506–755]	570 [439–683]	660 [523–784]	0.038
Blood transfusion, units	2 [1–3]	2 [1–4]	3 [1–3]	0.989
V-A ECMO pre-emptive	17 (37)	12 (50)	5 (23)	0.072
rescue	13 (28)	4 (17)	9 (41)	0.103
none	16 (35)	8 (34)	8 (36)	0.999
**Surgical prophylaxis**				
Carbapenems, *n* (%)	8 (17)	0 (0)	8 (36)	-
Ceftazidime-avibactam/ceftolozane-tazobactam ect, *n* (%)	10 (22)	0 (0)	14 (45)	-
β-lactam or III cephalosporins, *n* (%)	24 (52)	24 (100)	0 (0)	-
Colistin, fosfomycin, fluoroquinolones, *n* (%)	4 (9)	0 (0)	4 (18)^h^	-

Data are expressed as number and (percentage) or median and ) or median and [interquartile range]. ^h^: these antibiotics were used in combination with other antimicrobials. *Abbreviations*: LT, lung transplantation; V-A ECMO, venous-arterial extracorporeal membrane oxygenation

**Table 3:** Study outcomes according to surgical prophylaxis exposure

	Overall *N* = 46 (100)	Standard prophylaxis *N* = 24 (52)	Targeted prophylaxis *N* = 22 (48)	*p*-value
**Primary outcomes**	30 (65)	14 (58)	16 (73)	0.364
**Secondary outcomes**				
Infections by ESBL/MDR GN bacteria, *n* (%)	15 (33)	4 (17)^h^	11 (50)^h^	**0.027**
Colonizations by ESBL/MDR GN bacteria, *n* (%)	15 (33)	10 (42)	5 (23)	0.212
**Other outcomes**				
Infection by MS GN bacteria, *n* (%)	2(4)	1(4)	2(5)	0.999
Infection by GP bacteria, *n* (%)	6 (13)	2 (8)	4 (18)^l^	0.405
Invasive mechanical ventilation, hours	24 [20–59]	24 [19–64]	24 [20–59]	0.125
Re-tracheal intubation and/or tracheostomy, *n* (%)	10 (22)	2 (8)	8 (36)	**0.032**
Anastomotic complications, *n* (%)	4 (9)	2 (8)	2 (9)	0.999
30-day acute rejection^g^, *n* (%)	11 (24)	8 (33)	3 (14)	0.171
ICU LOS, days	7 [5–17]	6 [3–14]	8 [6–23]	**0.002**
Hospital LOS, days	33 [30–45]	33 [30–38]	35 [31–50]	**0.039**
Hospital mortality, *n* (%)	3 (7)	0 (0)	3 (14)	0.101
ECMO post-surgery, *n* (%)	5 (11)	3 (13)	2 (9)	0.999
PGD at 72 h	1 [0–2]	1 [0–1]	1 [0–2]	0.286
Immunosuppressive therapy (cyclosporine (*ref*))^m^	30 (65)	13 (54)	17 (77)	0.129
Renal replacement therapy, *n* (%))	6 (13)	2 (8)	4 (19)	0.405

*Data are expressed as number and (percentage) or median and [interquartile range]. ^h^: One patient treated with ‘standard’ prophylaxis and one patient with ‘targeted’ therapy, defined as ‘infected’ by MDR GN bacteria, had also a secondary colonization by different MDR GN bacteria; ^i^: one recipient, infected by a MS GN bacteria, was also colonized by a MDR GN bacteria. ^l^: one patients was colonized by postoperative MDR GN bacteria. ^g^: rejection is defined according to International Society for heart and lung transplantation (ISHLT) criteria (i.e., A3-A4 and/or B2 grade at biopsy) [38]. ^m^: The other LT recipients were treated with tacrolimus. *Abbreviations*: MDR, multidrug-resistant; MS, multisensitive; ICU, intensive care unit; LOS, length of stay; H, hospital; ICU, intensive care unit; ECMO, extracorporeal membrane oxygenation; PGD, primary graft dysfunction; *n*, number

Baseline characteristics of patients (collected from electronic health records) were summarized through descriptive statistics [number, proportion, median, interquartile range (IQR)]. Categorical variables were compared by chi-square (χ2), or Fisher exact test, when necessary. The Wilcoxon rank-sum test was used for the comparison of continuous variables. Statistical significance was defined as *p* values < 0.05. All analyses were conducted using R version 4.0.3 software (R Foundation for Statistical Computing) and Prism version 5.0 software (GraphPad Software, Inc.).

## Results

Overall, 181 patients underwent LT in our center between February 2016 and February 2023. Following eligibility criteria, 46 (25%) patients were included in the study (see study flow-chart in Fig. 1). Twenty-four (52%) pre-colonized recipients received ‘standard’ surgical prophylaxis, while 22 (48%) ‘targeted’ antibiotics. Baseline characteristics of both groups are described in Table 1. Twenty-four out of 46 (52%) enrolled patients were colonized by multisensitive GN bacteria and 22 (48%) by MDR GN bacteria. Most microorganisms were *Pseudomonas aeruginosa* and *Achromobacter xylosoxidans*, collected from respiratory samples (see full description of isolated bacteria in Table S1). Based on the available retrospective microbiological data from donors, 13 out of 46 (30%) donors tested positive on screening cultures, mostly from respiratory tract but never from blood stream (Table 1 and Table S1). β-lactam antibiotic or III cephalosporins were administered in all (100%) patients undergoing ‘standard’ prophylaxis; carbapenems, ceftazidime-avibactam, ceftolozane-tazobactam, colistin, fosfomycin, and fluoroquinolones were exclusively used in the ‘targeted’ group (Table 2).

### Prevalence of MDR GN bacteria after LT

**Fig. 2:**
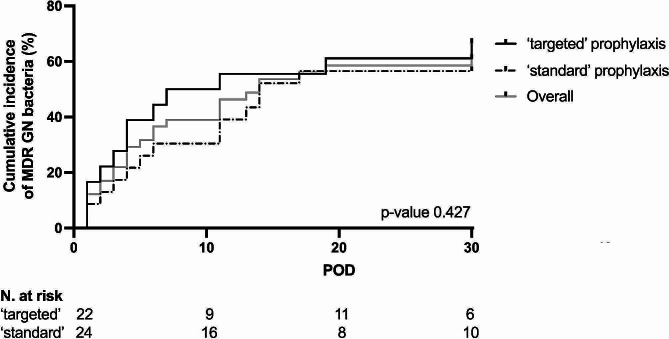
Cumulative incidence of postoperative MDR GN bacteria isolation according to surgical prophylaxis exposure. *Abbreviations*: LT, lung transplant; POD, postoperative day; MS, multisensitive; MDR, multidrug resistant

The overall prevalence of postoperative MDR GN bacteria isolation was 65% (30 patients) within the first 30 days after surgery (Table 3). The prevalence of postoperative MDR GN bacteria isolation was 58% (14 out of 24 patients) in the ‘standard’ prophylaxis group and 73% (16 out of 22 patients) in patients receiving ‘targeted’ therapy ( *p* = 0.364) (Table 3; Fig. 2).

Considering only LT recipients with postoperative MDR GN bacteria, 11 out of 14 (79%) patients treated with ‘standard’ group and 12 out of 16 (75%) exposed to ‘targeted’ therapies reconfirmed the same preoperative GN pathogen ( *p* = 0.999). The most frequent MDR GN bacteria isolates were *Pseudomonas aeruginosa* and *Klebsiella pneumoniae* from respiratory samples. More details on postoperative isolates are reported in Table S2.

### Prevalence of MDR GN bacterial infections and colonizations

According to Sepsis-3 criteria [25] and CDC/NHSN Surveillance Definitions for Specific Types of Infections [38] 15 out of 46 (33%) recipients developed infections due to MDR GN bacteria: nine patients (60%) were diagnosed with pneumonia, one patient (13%) with bacteremia, and four (27%) reported infections from multiple sites. Most of these recipients (11 out of 15) were exposed to ‘targeted’ surgical prophylaxis and only 4 to ‘standard’ antibiotics ( *p* = 0.027).

In 15 out of 46 (33%) patients, colonizations due to MDR GN bacteria were observed: in 9 patients (60%) positive samples derived from the airways, in three patients (39%) from the digestive tract, in one patient (13%) from the urinary tract, and in two patients (26%) from multiple sites. No differences were found between ‘standard’ vs. ‘targeted’ prophylaxis ( *p* = 0.217) (Table 3).

**Fig. 3:**
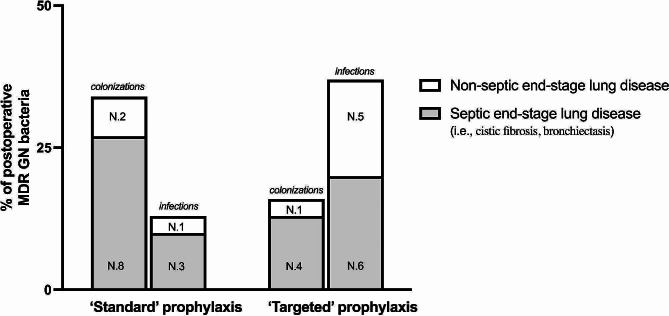
Infections and colonizations after LT based on exposure to different surgical prophylaxis. *Abbreviations*: LT, lung transplant; ns, non significant; *N*, number. *p*-value 0.427

As shown in Fig. 3, most (12 out of 15) colonizations and (9 out of 15) infections were recorded in recipients requiring LT due to septic end-stage lung diseases (*p* = 0.427).

### Other outcomes

Two (4%) recipients developed infections due to MS GN bacteria (*p*-value 0.999) and six (13%) patients due to GP bacteria (*p* = 0.405), with no differences between different prophylaxis (Table 3). Compared to patients exposed to the ‘standard’ prophylaxis, patients belonging to the ‘targeted’ group more frequently required re-tracheal intubation and/or tracheostomy (36% vs. 8%, *p* = 0.032), and recorded longer ICU and hospital stay (*p*-value 0.002 and 0.039, respectively) (Table 3).

## Discussion

In 46 bilateral LT recipients with previous GN-colonizations and not exposed to mechanical ventilation, ECMO or hospitalization before LT, the prevalence of postoperative MDR GN bacteria was 65% within the first 30 days after surgery. In the whole cohort, 33% of patients developed postoperative colonizations and 33% of recipients infections due to MDR GN bacteria. Interestingly, in patients exposed to ‘targeted’ antimicrobial prophylaxis the prevalence of postoperative infections due to MDR GN bacteria and the rate of re-intubations were higher, as well the ICU and hospital LOS were longer, compared to those treated according to ‘standard’ surgical prophylaxis. Finally, the postoperative occurrence of preoperative GN pathogens was similar between groups.

To the best of our knowledge, this is the first study exclusively including LT adult recipients pre-colonized by GN bacteria and describing the perioperative MDR GN bacterial epidemiology in this ‘specific’ patient population. Due to the remarkable worldwide increase of highly resistant pathogens, more data on the potential benefits of ‘personalized’ surgical antimicrobial prophylaxis are required. However, few studies have been published on this topic so far [7, 6, 24, 39–41].

According to the most recent literature, the rate of pre-operative GN colonization in LT recipients is still unclear [6, 7, 24, 40]. Recent findings suggest that the presence of preoperative MDR bacteria ranged from 1.1% to up to > 50% in recipients with cystic fibrosis [7, 20]. Our findings are in keeping with previous studies suggesting that previous antimicrobial treatments are key to the occurrence of highly resistant bacteria isolations after LT [6, 24, 40].

With regards to the occurrence of postoperative infection due to MDR GN bacteria, our prevalence of 33% is in line with previous investigations [7, 14]. However, these studies did not exclusively enroll pre-colonized LT recipients, but patients receiving solid organ transplants with or without pre-existing bacterial isolations [6, 14], thus highlighting that previous recipient-related colonizations are not necessarily associated with a greater risk of infection [6, 14].

We reported a similar prevalence between postoperative colonizations (33%) and infections (33%) due to MDR GN bacteria. Noteworthy, we observed that the exposure to a ‘targeted’ antimicrobial surgical prophylaxis was associated with increased risk of postoperative infections by highly resistant bacteria, especially in recipients needing LT due to septic end-stage lung diseases (i.e., cystic fibrosis or bronchiectasis). This finding is in keeping with Boscolo et al. and Paglicci et al., who reported that the use of ‘standard’ antibiotic prophylaxis, when compared to broad-spectrum antibiotic regimens, reduced the incidence of highly resistant bacteria infection after LT [6, 7]. Moreover, the ‘targeted’ prophylaxis did not prevent the occurrence of the preoperative GN bacteria during the first month after surgery [7, 14].

Our preliminary findings may suggest that antimicrobial surgical prophylaxis titrated on preoperative GN bacteria colonizations in LT recipients, at low-risk of postoperative complications, might promote the selection of resistant bacteria, increasing the risk of postoperative infections, and affect patient outcomes. The last updated guidelines from the American Society of Transplantation and European Society of Clinical Microbiology and Infectious Diseases confirmed that heightened infection control and antimicrobial stewardship initiatives are needed to prevent these ‘difficult-to-treat’ infections, curtail their transmission, and limit the evolution of MDR GN pathogens. Despite these recommendations, no clear information has been described about the use of ‘personalized’ perioperative prophylaxis but a strong recommendation has been reported about the sparing of carbapenems and other antibiotics for the risk to select resistant microorganisms [21]. The International consensus recommendations for anesthetic and intensive care management of lung transplantation, released in 2022 and keeping in line with our findings, confirmed that in uncomplicated LT recipients with low risk for donor and recipient-derived infection, as those patients enrolled in our study, a short antibacterial prophylaxis, primarily aimed at preventing surgical site infections, should be administered (*strong consensus*). Noteworthy, in case of positive cultures, postoperative antimicrobial treatment should be modified according to the isolated microorganism and the risk of postoperative infections [22]. On the contrary, the last Spanish guidelines, albeit with *weak evidence*, are in favor of personalized antibiotic prophylaxis based on the pre-existing colonization. However, the same authors underlined the importance to balance the risk of infection against the risk of developing adverse effects to the antibiotics and/or carbapenem-resistance [23].

Our study has some limitations. First, it is a retrospective monocentric observational study which needs to be confirmed by well-designed randomized clinical trials without biases related to a ‘non-standardized’ antimicrobial prescription. The retrospective nature of the study did not allow to investigate the reasons of targeted prophylaxis, potentially related to the patient’s clinical history and baseline characteristics at the time of transplant. Second, microbiological data and morbidity rate were only investigated within 30 days after LT and not later. However, many studies have found a greater occurrence of MDR bacteria within the first month following surgery [5, 6]. Third, we did not report in our study cohort, frequent occurrence of viral infection or precolonized, well-known risk factors of morbidity in LT recipients, as suggested by literature data [42].

Moreover, the small sample size did not allow us to perform any multivariable regression analysis or to apply a Propensity Score methodology for minimizing potential differences between sub-groups. Therefore, our results barely suggest an association between the choice of antimicrobial surgical prophylaxis and the analyzed outcomes in LT patients, without excluding potential selection biase. Lastly, we excluded recipients with pre-colonizations due to GP bacteria, as their impact seems to be declining in solid organ transplant recipients [18, 34].

## Conclusions

In our cohort of 46 bilateral LT recipients with preoperative GN bacteria, the occurrence of postoperative isolations of MDR GN bacteria was frequent (65%) but not necessarily associated with a high risk of postoperative infection. In fact, a prophylactic antibiotic approach tailored to preoperative colonizations was mostly associated with an increased prevalence of postoperative MDR GN bacterial infections and worse short- and mid-term clinical outcomes, as compared to a ‘standard’ prophylaxis.

### Electronic supplementary material

Below is the link to the electronic supplementary material.

Supplementary Material 1

## Data Availability

“The datasets used and/or analysed during the current study available from the corresponding author on reasonable request”.

## Abbreviations

LT: Lung transplant
IMV: Invasive mechanical ventilation
ECMO: Extracorporeal membrane oxygenation
GP: Gram-positive
