# Supplementary material for: Gram-negative bacterial colonizations before bilateral lung transplant. The impact of ‘targeted’ versus ‘standard’ surgical prophylaxis
Source: BMC Infect Dis. 2024 Mar 13;24:307. doi: 10.1186/s12879-024-09199-y (PMC10935849; doi:10.1186/s12879-024-09199-y)
Supplement: Supplementary file 1 — Supplementary Material 1 [file 12879_2024_9199_MOESM1_ESM.docx]

**SUPPLEMENTARY DATA**

**Title: Gram-negative bacterial colonizations before bilateral lung transplant. The impact of ‘targeted’ versus ‘standard’ surgical prophylaxis.**

**Sabrina Congedi ^1^, Arianna Peralta ^2^, Luisa Muraro ^2^, Martina Biscaro ^1^, Tommaso Pettenuzzo ^2^, Nicolò Sella ^2,^*, Silvia Crociani ^1^, Arméla Anne-Sabine Tagne ^1^, Ida Caregnato ^1^, Francesco Monteleone ^1^, Elisa Rossi ^2^, Gabriella Roca ^3^, Silvia Manfrin ^1^, Serena Marinello ^4^, Maria Mazzitelli ^4^, Andrea Dell’Amore ^3^, Annamaria Cattelan ^4^, Federico Rea ^3^, Paolo Navalesi ^1,2^, Annalisa Boscolo ^1,2,3^**

**Table S1. Previous colonizations in LT recipients and donors. Pag. 2**

**Table S2. MDR GN bacteria within 30 days following LT. Pag. 4**

**Table S3. STROBE Statement—Checklist. Pag. 5**

**Table S4. Microbiological surveillance for bacteria (protocol). Pag. 10**

**Table S1. Previous colonizations in LT recipients and donors.**

| **Pre-colonized RECIPIENTS*** | | Total N. 46 | **Pre-colonized DONORS°** | | Total N. 13 | |
| --- | --- | --- | --- | --- | --- | --- |
| **GN bacteria** | | | **GN bacteria** | | | |
|  | *Pseudomonas aeruginosa* | 34 |  | *Pseudomonas aeruginosa* | | 1 |
|  | *Klebsiella pneumoniae* | 4 |  | *Klebsiella pneumoniae* | | - |
|  | *Enterobacter cloacae* | 1 |  | *Klebsiella oxytoca* | | 2 |
|  | *Enterobacter aerogenes* | 2 |  | *Enterobacter aerogenes* | | 2 |
|  | *Proteus mirabilis* | 1 |  | *Enterobacter cloacae* | | 3 |
|  | *Escherichia coli* | - |  | *Citrobacter freundii* | | 1 |
|  | *Haemophilus influenzae* | 4 |  | *Citrobacter braakii* | | - |
|  | *Haemophilus haemolyticus* | - |  | *Haemophilus influenza* | | 2 |
|  | *Achromobacter xylosoxidans* | 11 |  | *Serratia marcescens* | | 1 |
|  | *Stenotrophomonas maltophilia* | 3 |  | *Escherichia coli* | | 1 |
|  | *Hafnia alvei* | 1 |  |  | |  |
| **GP bacteria** | | | **GP bacteria** | | | |
|  | *Staphylococcus aureus* | 19 |  | *Staphylococcus aureus* | | 1 |
|  | *Staphylococcus epidermidis* | - |  | *Streptococcus pyogenes* | | - |
|  | *Streptococcus viridans*  *Streptococcus agalactiae* | -  - |  |  | |  |
| **Fungi** | | | **Fungi** | | | |
|  | *Candida spp* | 2 |  | *Candida spp* | | 1 |
|  | *Aspergillus spp* | 2 |  | *Aspergillus spp* | | - |

Data are expressed as the number of pre-colonized donors or recipients. *In recipients: *Pseudomonas aeruginosa* and *Achromobacter xylosoxidans* were exclusively isolated from airways samples. Among GP bacteria, 5 *Staphylococcus aureus* were methicillin-resistant. °: all positive cultures were obtained from respiratory airways or rectal swabs. No samples were collected from the blood stream.

*Abbreviations:* GN, Gram-negative; GP, gram-positive. MDR, multidrug-resistant; LT, lung transplant; N, number; spp: species.

**Table S2. MDR GN bacteria within 30 days following LT.**

| **MDR GN bacteria** | | | **N. of recipients** | **Biological samples (N. of recipients)** | | | | | |
| --- | --- | --- | --- | --- | --- | --- | --- | --- | --- |
|  |  | Total N. 30 (65) | |  | ***Digestive tract (rectum swab)*** | ***Respiratory airways*** | ***Blood stream*** | ***Urine*** | ***Surgical wound/drainage*** |
|  | *Pseudomonas aeruginosa* | 15 (50) | |  | 1 | 14 | 1 | - | 1 |
|  | *Klebsiella pneumoniae*  *Escherichia coli* | 10 (33)  5 (17) | |  | 5 | 5  5 | 1  - | 2  - | 1  - |
|  | *Enterobacter cloacae* | 1 (3) | |  |  | 1 | - | - |  |
|  | *Stenotrophomonas maltophilia* | 1 (3) | |  | - | 1 | - | - | - |
|  | *Achromobacter xylosoxidans* | 2 (7) | |  | - | 2 | - | - | - |

Values are expressed as the number of LT recipients with MDR GN bacteria or as (percentage). *Abbreviations:* GN, Gram-negative; MDR, multidrug-resistant; LT, lung transplant; N, number.

**Table S3. STROBE Statement—Checklist.**

|  | **Item No** | **Recommendation** | **Page No** |
| --- | --- | --- | --- |
| **Title and abstract** | 1 | (*a*) Indicate the study’s design with a commonly used term in the title or the abstract | 1 |
|  |  | (*b*) Provide in the abstract an informative and balanced summary of what was done and what was found | 1 |
| **Introduction** | | | |
| Background/rationale | 2 | Explain the scientific background and rationale for the investigation being reported | 2 |
| Objectives | 3 | State specific objectives, including any prespecified hypotheses | 2 |
| **Methods** | | | |
| Study design | 4 | Present key elements of study design early in the paper | 9 |
| Setting | 5 | Describe the setting, locations, and relevant dates, including periods of recruitment, exposure, follow-up, and data collection | 9 |
| Participants | 6 | (*a*) Give the eligibility criteria, and the sources and methods of selection of participants. Describe methods of follow-up | 10 |
|  |  | (*b*) For matched studies, give matching criteria and number of exposed and unexposed | - |
| Variables | 7 | Clearly define all outcomes, exposures, predictors, potential confounders, and effect modifiers. Give diagnostic criteria, if applicable | 10 |
| Data sources/ measurement | 8* | For each variable of interest, give sources of data and details of methods of assessment (measurement). Describe comparability of assessment methods if there is more than one group | 10 |
| Bias | 9 | Describe any efforts to address potential sources of bias | - |
| Study size | 10 | Explain how the study size was arrived at | Figure 1 |
| Quantitative variables | 11 | Explain how quantitative variables were handled in the analyses. If applicable, describe which groupings were chosen and why | 10 |
| Statistical methods | 12 | (*a*) Describe all statistical methods, including those used to control for confounding | 10 |
|  |  | (*b*) Describe any methods used to examine subgroups and interactions | 10 |
|  |  | (*c*) Explain how missing data were addressed | - |
|  |  | (*d*) If applicable, explain how loss to follow-up was addressed | - |
|  |  | (*e*) Describe any sensitivity analyses | - |
| **Results** | | |  |
| Participants | 13* | (a) Report numbers of individuals at each stage of study—eg numbers potentially eligible, examined for eligibility, confirmed eligible, included in the study, completing follow-up, and analysed | 3 |
|  |  | (b) Give reasons for non-participation at each stage | - |
|  |  | (c) Consider use of a flow diagram | Figure 1 |
| Descriptive data | 14* | (a) Give characteristics of study participants (eg demographic, clinical, social) and information on exposures and potential confounders | 3 |
|  |  | (b) Indicate number of participants with missing data for each variable of interest | - |
|  |  | (c) Summarise follow-up time (eg, average and total amount) | - |
| Outcome data | 15* | Report numbers of outcome events or summary measures over time | 3-6 |
| Main results | 16 | (a) Give unadjusted estimates and, if applicable, confounder-adjusted estimates and their precision (eg, 95% confidence interval). Make clear which confounders were adjusted for and why they were included  (b) Report category boundaries when continuous variables were categorised  (c) If relevant, consider translating estimates of relative risk into absolute risk for a meaningful time period | 3-6 |
| Other analyses | 17 | Report other analyses done—eg analyses of subgroups and interactions, and sensitivity analyses | - |
| **Discussion** |  |  |  |
| Key results | 18 | Summarise key results with reference to study objectives | 8 |
| Limitations | 19 | Discuss limitations of the study, taking into account sources of potential bias or imprecision.  Discuss both direction and magnitude of any potential bias | 9 |
| Interpretation | 20 | Give a cautious overall interpretation of results considering objectives, limitations, multiplicity of analyses, results from similar studies, and other relevant evidence | 9 |
| Generalisability | 21 | Discuss the generalisability (external validity) of the study results | - |
| **Other information** |  |  |  |
| Funding | 22 | Give the source of funding and the role of the funders for the present study and, if applicable, for the original study on which the present article is based | - |

**Table S4. Microbiological surveillance for bacteria (protocol).**

|  | ***Rectum*** | ***Respiratory airways***^c^ | ***Blood stream*** | ***Urine*** | ***Surgical wound/others*** |
| --- | --- | --- | --- | --- | --- |
| **At ICU admission** | yes | yes | - | - | yes |
| **During ICU stay** | twice a week  (*monday-thursday*) | every 2^a^-3^b^ days | twice a week  (*monday-thursday*) | twice a week  (*monday-thursday*) | based on clinical need |
| **After ICU discharge** | once a week | every 3-5 days | one a week | once a week | based on clinical need |

^a^: during invasive mechanical ventilation; ^b^: during spontaneous breathing; ^c:^bronchoaspiration/bronchoalveolar lavage (recommended). *Abbreviations:* ICU: intensive care unit.
